# Identification of biomarkers related to neutrophils and two molecular subtypes of systemic lupus erythematosus

**DOI:** 10.1186/s12920-022-01306-9

**Published:** 2022-07-20

**Authors:** Huiyan Li, Pingting Yang

**Affiliations:** Department of Rheumatology and Immunology, First Affiliated Hospital, China Medical University, Shenyang, 110001 China

**Keywords:** Autoimmune disease, Systemic lupus erythematosus, Biomarker, Immunotherapy

## Abstract

**Background:**

Systemic lupus erythematosus (SLE), an autoimmune disease with complex pathogenesis, poses a considerable threat to women’s health. Increasing evidence indicates that neutrophils play an important role in the development and progression of lupus.

**Methods:**

Weighted correlation network analysis and single-sample gene set enrichment analysis (GSEA) were used to analyse SLE expression data from a comprehensive gene expression database and identify modules associated with neutrophils. Thereafter, the biomarkers most closely related to neutrophils were identified. We reclassified SLE into two molecular subtypes based on the aforementioned biomarkers and evaluated cell infiltration, molecular mechanisms, and signature pathways in each subtype.

**Results:**

The results showed significant differences in immunological characteristics between the two molecular subtypes of SLE. Hub genes were significantly upregulated in the NEUT-H subtype, and they may be associated with lupus activity. The GSEA revealed associations between our biomarkers and key metabolic pathways.

**Conclusions:**

Our study provides not only a classification for patients with SLE but also new cell and gene targets for immunotherapy, as well as a new experimental paradigm to explore immunotherapy for other autoimmune diseases.

**Supplementary Information:**

The online version contains supplementary material available at 10.1186/s12920-022-01306-9.

## Background

Systemic lupus erythematosus (SLE) is a multi-system autoimmune disease caused by congenital and adaptive immune system disorders, including clonal amplification of autoreactive lymphocytes, production of autoantibodies, and increased production of a variety of cytokines and other inflammatory mediators [1]. Driven by genetic and environmental factors, the vicious cycle of autoantigen exposure, autoantibody production, chronic inflammation, and tissue damage leads to SLE occurrence [2, 3]. Improperly activated neutrophils are most likely to cause damage to local tissues, because they are present in large numbers at the site of inflammation and their cytotoxic contents are released directly onto host tissues [1]. Neutrophil-derived reactive oxygen species (ROS) and granuloproteinases are associated with vascular tissue damage and post-translational modifications of pathogenic DNA and proteins in SLE [1]. The imbalance of neutrophil energy metabolism leads to SLE, and impaired reduction–oxidation capacity increases ROS-mediated damage in SLE [4]. Neutrophil extracellular traps (NETs) are net-like structures composed of DNA-histone complexes and proteins released by activated neutrophils, formed by the combination of cytoplasm and granular proteins [5]. Recently, increasing evidence has shown that neutrophil dysregulation in patients with SLE is closely related to disease severity and prognosis [6]. NETs induce endothelial injury, promote thrombosis, and contain sources of autoantigens that trigger autoimmunity [7]. In addition, patients with SLE are characterised by the presence of specific subsets of circulating low-density granulocytes (LDG) that are phenotypically, functionally, and transcriptionally distinct from other subsets of neutrophils, and LDGs are highly proinflammatory [8].

To date, SLE classification and diagnosis have relied on clinical manifestations and autoimmune serology rather than molecular approaches [9]. Therefore, molecular changes in these heterogeneous autoimmune diseases should be further explored, and new classification systems have been established to develop more rational, specific, and effective treatments.

An increasing number of studies have applied high-throughput techniques to reveal the molecular mechanisms of SLE incidence or progression. However, most of them have focused on the differences between patients with SLE and healthy controls [10], while only a few studies have investigated the differences between patients with SLE. Moreover, molecular typing of SLE neutrophils in the background of immune-related genes and the clinical significance of related markers are currently lacking. In this study, bioinformatic analysis was used to combine a relatively homogeneous population of patients with SLE, and consistent cluster analysis of SLE was performed based on neutrophil enrichment. Two subtypes were identified, characterised by significantly different immune infiltration, molecular mechanisms, signature pathways, and drug sensitivities. This study not only revealed neutrophil biomarkers in patients with SLE but also provided a new perspective for SLE treatment.

## Methods

### Microarray data

The gene expression profiles of the datasets GSE100163, GSE102466, GSE121239, and GSE126307 were screened and downloaded from the Gene Expression Omnibus (GEO) database of the National Center for Biotechnology Information, which is free and publicly available [11]. We selected GSE100163, which included a total of 69 whole blood samples, of which 55 were samples of patients with SLE and 14 were healthy control samples. Thirty-one and nine samples of peripheral blood mononuclear cells (PBMCs) from patients with SLE and healthy human controls (PBMC) were included in GSE126307. GSE102466 contained 26 whole blood samples of patients with SLE. GSE121239 comprised 20 and 292 PBMC samples from healthy humans and patients with SLE, respectively. As we focused only on samples of patients with SLE, normal samples were excluded.

GSE100163: Illumina HumanWG-6 v3.0 expression beadchip

GSE126307: Illumina Human Whole-Genome DASL HT

GSE102466: Affymetrix Human Genome U219 Array

GSE121239: [HT_HG-U133_Plus_PM] Affymetrix HT HG-U133 + PM Array Plate

### Screening of immune-related genes

From the Immunology Database and Analysis Portal (ImmPort; https://immport.niaid.nih.gov) [12], more than 1671 immune-related genes from a complete list were downloaded. These genes are involved in the functions of multiple immune-related pathways, including T and B cell receptor signalling pathways and antigen processing and rendering; encoding chemokines, interleukins, interleukin receptors, chemokine receptors, cytokines, interferons, interferon receptors, cytokine receptors, tumour necrosis factor (TNF), TNF family member receptor, transforming growth factor-β (TGF-β) family member, and TGF-β family member receptor; and natural killer cell cytotoxicity.

### Single-sample gene set enrichment analysis to quantify the relative neutrophil abundance

Single-sample gene set enrichment analysis (ssGSEA) was used to sequence the genes according to their absolute expression in the samples [13]. The ssGSEA was used to determine the enrichment scores of different immune cell subsets, related functions, or pathways. The relative abundance of each immune cell type is expressed by the enrichment fraction in the ssGSEA and normalised as a uniform distribution from 0 to 1 [14]. A set of genes with specific characteristics used to label each immune cell type was obtained from recent studies, and the biosimilarity of infiltrated immune cells was estimated by multidimensional scaling and a Gaussian fitting model. Adaptive immune cells included activated B cells, activated CD8 T cells, effector memory CD8 T cells, and activated CD4 T cells, whereas innate immune cells included eosinophils, activated dendritic cells, immature dendritic cells, natural killer T cells, and neutrophils.

### Weighted correlation network analysis

Weighted correlation network analysis (WGCNA) is a systems biology method used to describe the correlation patterns between genes [15]. In recent years, this analysis has been increasingly used [16]. In this study, WGCNA was used to identify gene modules highly correlated with traits for the subsequent analysis. Module Eigengene (ME) refers to the first principal component of a given Module and is considered to represent the gene expression profile of a given gene module. Using correlation analysis of gene expression and ME, the value of the MODULE Membership (MM) was obtained. The MM value was essentially the Pearson correlation coefficient between the gene and module. When the absolute value of MM was close to 1, it indicated that the genes were highly correlated with the Module. The correlation analysis was conducted between the expression level of a specific gene and the corresponding phenotypic value. The final value of the correlation coefficient was gene significance (GS), which reflected the correlation between the gene expression level and phenotypic data. The higher the GS, the greater the correlation between the specified gene and the study phenotype [17, 18]. Genes in key modules, MM < 0.5 and GS < 0.5, were defined as hub genes.

### Gene Ontology and Kyoto Encyclopedia of Genes and Genomes pathways

Gene Ontology (GO) is an international gene functional classification system that describes three properties of a gene, namely, the biological process (BP) involved, the product function of the gene, and the spatial localisation of the gene product within organelles [19]. It also describes the distribution of molecular functional enrichment (MF) in the cell component (CC) of BPs [6]. To further analyse the BPs of neutrophil-associated core genes, GO annotation and Kyoto Encyclopedia of Genes and Genomes (KEGG) pathway enrichment analyses of these genes were performed the Database for Annotation, Visualisation and Integrated Discovery (DAVID 6.8, https://david.ncifcrf.gov/. *P*-values < 0.01 and a false discovery rate (FDR) < 0.01 were used as the enrichment and screening conditions, and confidence was built using the Search Tool for the Retrieval of Interacting Genes/Proteins (https://david.ncifcrf.gov/) and CytoScape version 3.7.2. The protein–protein interaction (PPI) network had a confidence coefficient of 0.4.

### Identification of SLE subtypes based on neutrophil gene sets

For each SLE dataset, the ssGSEA was performed using Gene Set Variation Analysis kits to quantify the enrichment level of each SLE sample in the neutrophil-characteristic gene set. The ConsensusClusterPlus package was used for consensus clustering and neutrophil subtype screening for ssGSEA scoring. Briefly, k-means clustering was performed using 50 iterations (with 80% of each sample). The optimal cluster number was determined using the cluster fraction of the cumulative distribution function (CDF) curve, and the relative change in the area under the CDF curve was evaluated.

### Heatmap

The ssGSEA score xi of each SLE sample was converted to XI', using the following formula:

1 $${\text{x}}_{{\text{i}}}^{{\prime }} \, = \, \left( {{\text{x}}_{{\text{i}}} - {\text{ x}}_{{{\text{min}}}} } \right) \, / \, \left( {{\text{x}}_{{{\text{max}}}} - {\text{ x}}_{{{\text{min}}}} } \right)$$

where x_max_ and x_min_ represent the maximum and minimum ssGSEA scores of all samples in the SLE dataset, respectively. The PheatMap package in R was used for heatmap visualisation.

### Assessment of immune cell infiltration in SLE

In this study, the R package ‘CIBERSORT’ was used to estimate the proportion of immune cells in the GSE100163, GSE102466, GSE121239, and GSE126307 datasets. Specifically, the CIBERSORT algorithm was used to calculate the scores of 22 SLE types [20]. CIBERSORT is a tool developed at Stanford University to deconvolute the expression matrix of human leukocyte subtypes [20]. The principle of linear support vector regression was used for deconvolution analysis of the expression matrix of unknown mixtures containing similar cell types; therefore, it is a useful tool to estimate the abundance of specific cells in mixed tissues. Next, the corrplot software package [21] was used to draw the relevant heat map to visualise the correlation of 22 types of infiltrated immune cells. The ggplot2 software package [22] was used to draw the violin diagram to visualise the differences in immune cell infiltration.

### Verification of immune correlation

To verify the immune correlation between neutrophil-associated biomarkers, Pearson correlation analysis was used to analyse the correlation between these markers and 22 immune cells. The correlation index R and the corresponding *P*-value were visualised using the ggplot2 and DPLYR packages.

### Correlation of multiple immune genes

The correlation among eight biomarkers was explored, and Pearson correlation analysis was used to analyse the correlation between the above genes. Corrplot and Corrgram in R language were used for visualisation. Red and blue represent positive and negative correlations, respectively.

### GSEA

Biological changes in each of the two subtypes in the GEO dataset were compared. C5.all.v7.4.symbol.GMT (Gene Ontology) and C2.cp.kegg.v7.4.symbol [curated] were used as reference gene sets for GSEA. Using 1000 permutations, an error detection rate (FDR) < 0.05 was used as the screening threshold, and GSEA version 4.0.1 was used for analysis.

### Drug sensitivity analysis

The CellMiner database (https://discover.nci.nih.gov/cellminer/home.do) was used to download drug susceptibility gene expression data samples, after clinical laboratory validation, and Food and Drug Administration (FDA) certification standards for screening susceptibility data. Thereafter, the expression of neutrophil-related biomarkers was combined with drug sensitivity data for Pearson correlation analysis. Finally, the correlation between neutrophil-related gene expression and drug sensitivity was obtained.

## Results

### ssGSEA and WGCNA quantified neutrophils

**Fig. 1:**
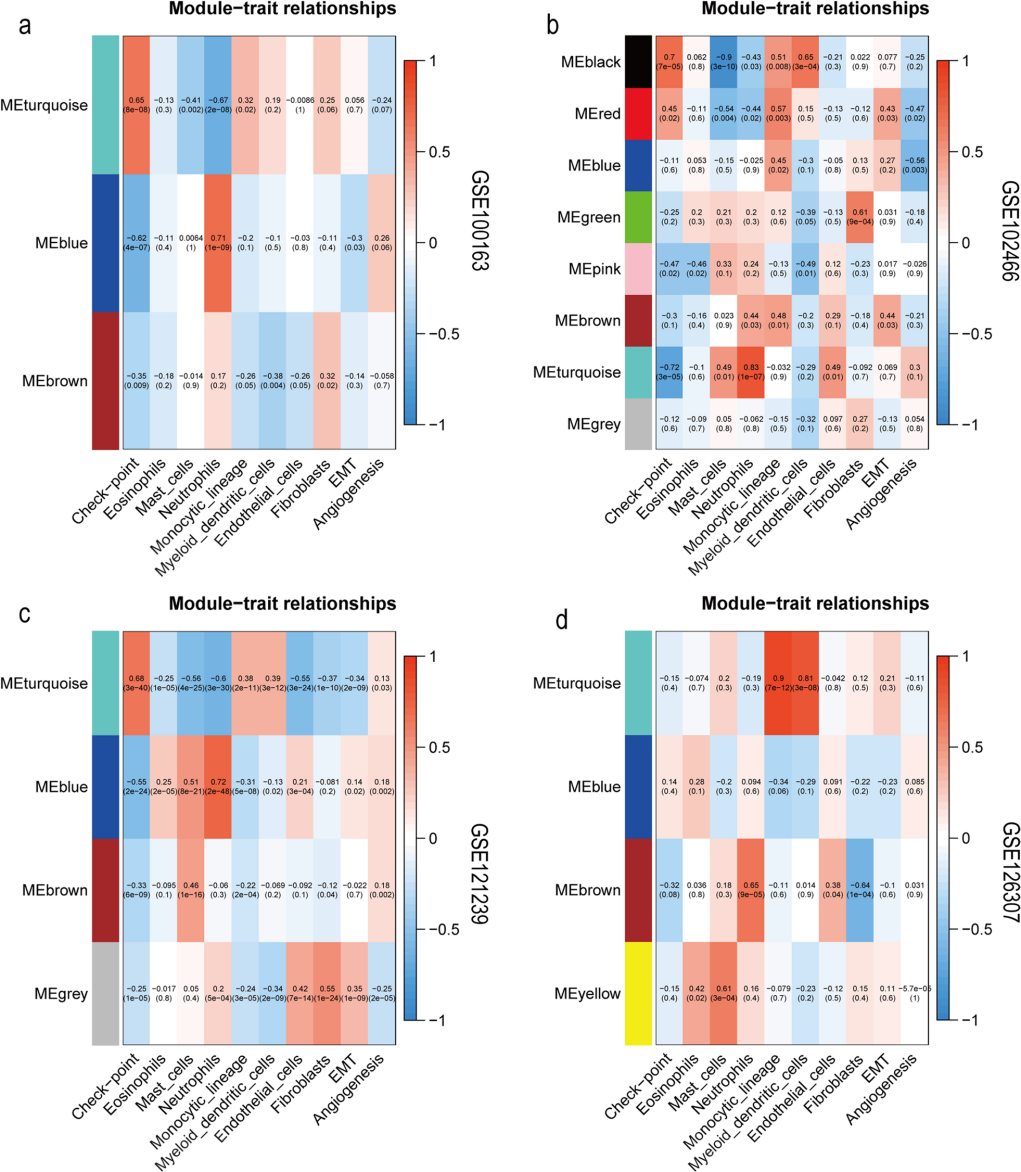
Identification of key genes based on WGCNA for SLE neutrophil subtypes. **a–d** Module–trait relationships in SLE

First, a specific set of characteristic genes for labelling each immune cell type was obtained from a recent study [23]. Quantification results of neutrophils in innate immune cells were selected, and 1671 immune genes were downloaded from the IMMPORT database for gene co-expression network analysis (WGCNA). In Fig. 1, each cell contains the corresponding correlation and *P* values. The larger the positive value, the stronger the correlation.

1. Three gene modules were obtained from GSE100163. The blue module positively correlated with neutrophils, and the blue–green module negatively correlated with neutrophils. The blue module comprised 423 neutrophil genes.
2. Eight gene modules were obtained from GSE102466. Brown, pink, and blue–green modules positively correlated with neutrophils, and the blue–green module showed the highest correlation, containing 292 neutrophil-related genes.
3. Four gene modules were obtained from GSE121239. The blue module positively correlated with neutrophils and had the highest correlation, corresponding to 156 neutrophil-related genes.
4. Four gene modules were obtained from GSE126307. The brown module positively correlated with neutrophils and had the highest correlation, corresponding to 118 neutrophil-related genes.

**Fig. 2:**
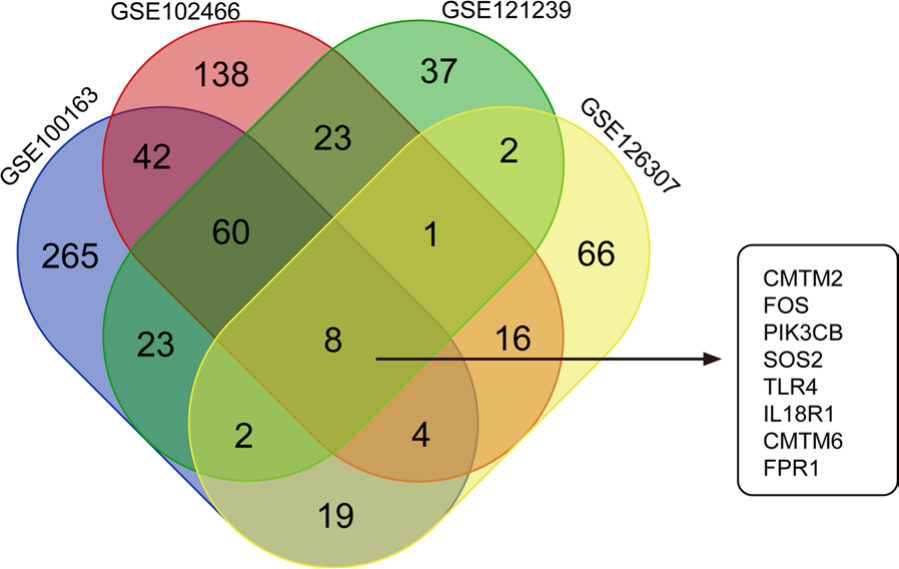
The result of the shared genes. The shared genes between the blue module of GSE100163, the blue–green module of GSE102466, the blue module of GSE121239, and the brown module of GSE126307 by overlapping them

A Venn diagram (Fig. 2) was drawn for the neutrophil-related genes in the four gene sets, and eight biomarkers with the highest correlation with neutrophils were obtained for the subsequent analysis. These included chemokine-like factor (CKLF)-like MARVEL transmembrane domain containing 2 (*CMTM2*), Fos proto-oncogene (*FOS*), phosphatidylinositol-4,5-bisphosphate 3-kinase catalytic subunit beta (*PIK3CB*), SOS Ras/Rho guanine nucleotide exchange factor 2 (*SOS2*), Toll-like receptor 4 (*TLR4*), (interleukin 18 receptor 1 (*IL18R1*), *CMTM6*, and formyl peptide receptor 1 (*FPR1*).

### Identification of two molecular subtypes of SLE based on neutrophil hub genes

**Fig. 3:**
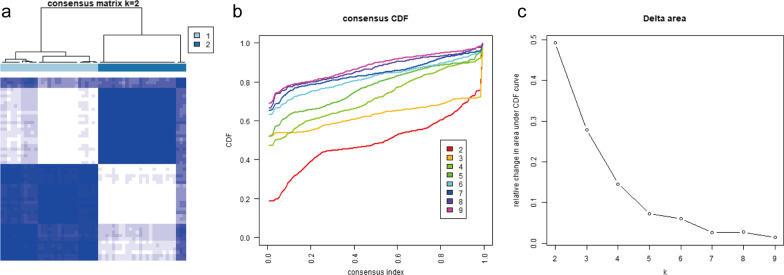
Consistent clustering was performed for SLE. **a** Heat map of sample clustering at consensus, k = 2. **b** Cumulative distribution function (CDF) curve. **c** CDF delta area curve of consensus clustering indicating the relative change gin area under the CDF curve for each category number k compared with k − 1

**Fig. 4:**
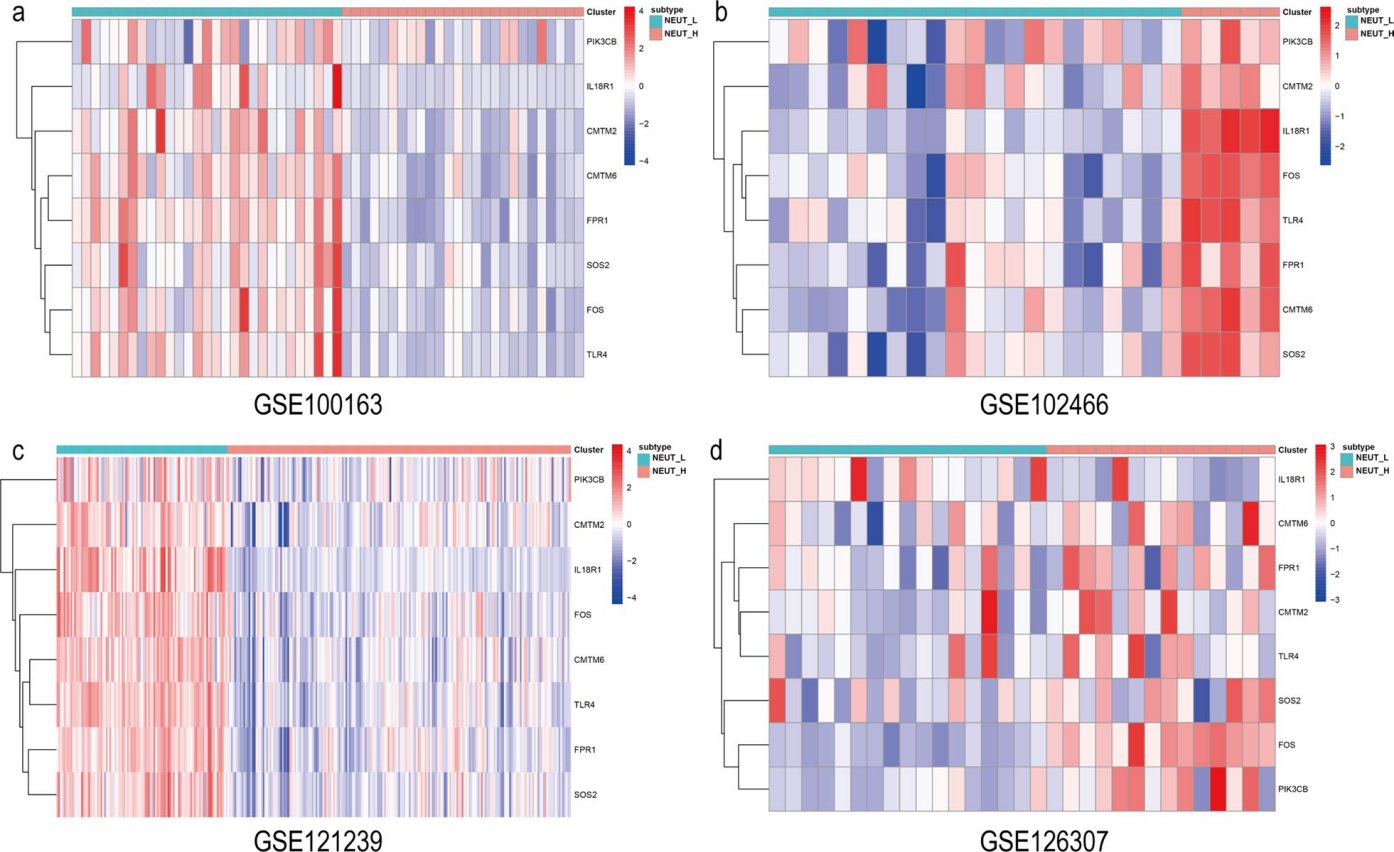
Expression of neutrophil-related genes in the SLE subtype. **a** GSE100163, **b** GSE102466, **c** GSE121239, **d** GSE126307

Based on consistent clustering, SLE was divided into different subgroups based on the expression of neutrophil-related gene characteristics. The ConsensusClusterPlus package was used to classify all SLE samples into K (k = 2–9) distinct subtypes. Figure 3b shows CDF distribution under different cluster numbers k, and the higher the value, the more stable the clustering result under this value. The delta area diagram, in Fig. 3c, shows the relative changes in areas under the CDF curve between k and k-1. A higher value indicates that the clustering effect under this value has a more obvious improvement in the goodness of clustering effect compared with k-1. Overall, when k = 2, the CDF curve based on consensus score achieved optimal partition (Fig. 3), defined as NEUT_H and NEUT_L. Similarly, the same clustering and subtyping for datasets GSE100163, GSE102466, GSE121239, and GSE126307 were performed (Fig. 4).

### Results of immune cell infiltration

**Fig. 5:**
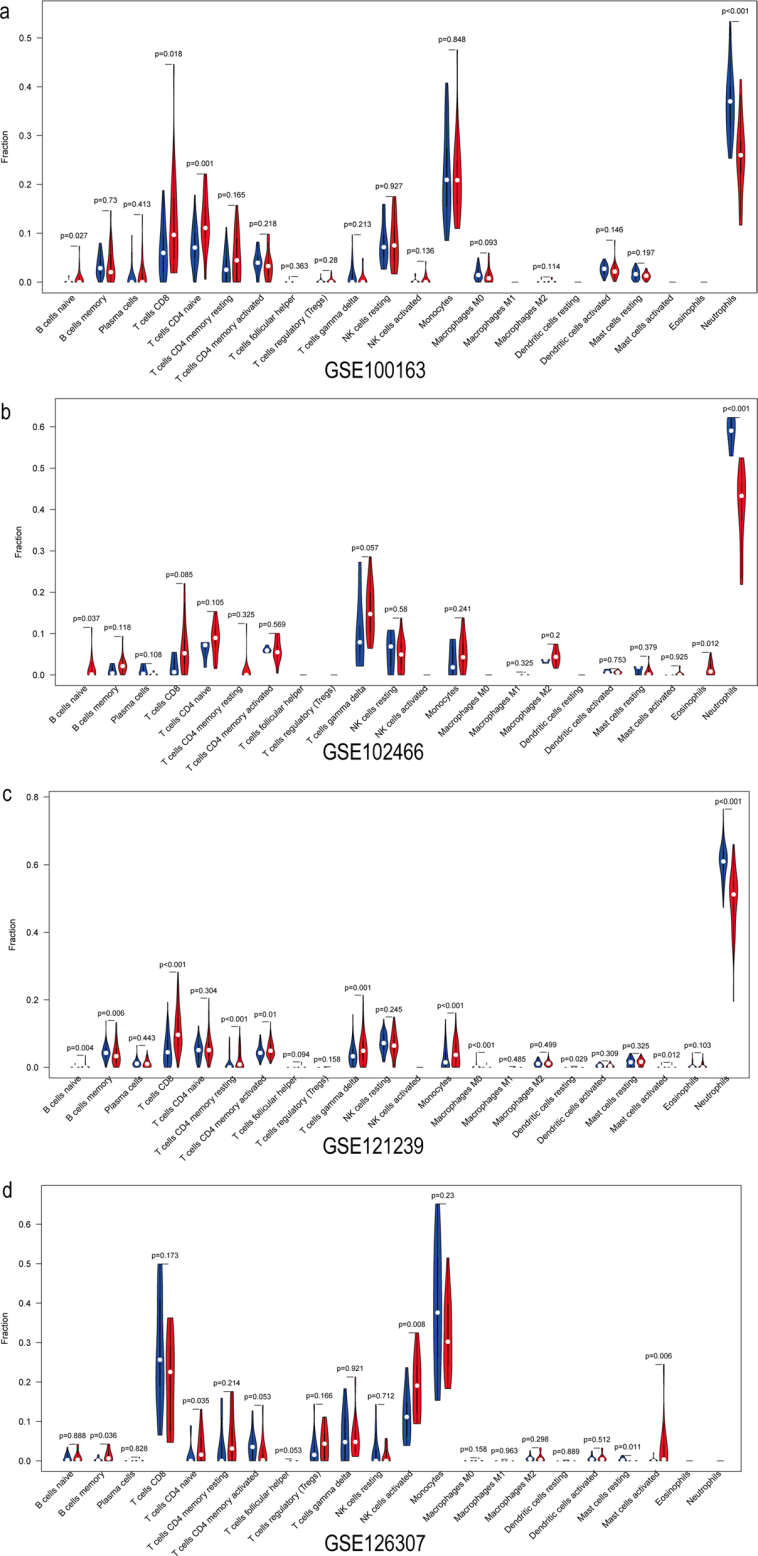
Violin diagram of the proportion of 22 types of immune cells. **a** GSE121239, **b** GSE126307, **c** GSE121239, **d** GSE126307showed the difference in infiltration between the two clusters

First, the infiltration of 22 immune cell subsets in two SLE molecular classifications was revealed, and then CIBERSORT was used to investigate the differences between high and low neutrophil-enriched groups. The violin diagram in Fig. 5 shows that the proportion of immune cells varies significantly between the groups. Compared with that of the low neutrophil enrichment group, neutrophil infiltration in GSE100163 was high (*P* < 0.001), and infiltration of naive B cells (*P* < 0.027) and CD8 T cells (*P* = 0.018) was low in the high immunogroup. In GSE102466, there was greater infiltration of neutrophils (*P* < 0.001) and less infiltration of basophils (*P* = 0.012) and naive B cells (*P* = 0.037). In GSE121239, the infiltration of neutrophils (*P* = 0.001) and B cell memory cells (*P* = 0.006) was higher than that of naive B cells (*P* = 0.004) and CD8 T cells (*P* < 0.001). No significant correlation between cell infiltration was found in the GSE126307 dataset.

#### Validation of immunological association

The eight identified genes are potential immunotherapeutic targets, and their relationship and interaction with immune cells are of significance for further immune-related studies.

**Fig. 6:**
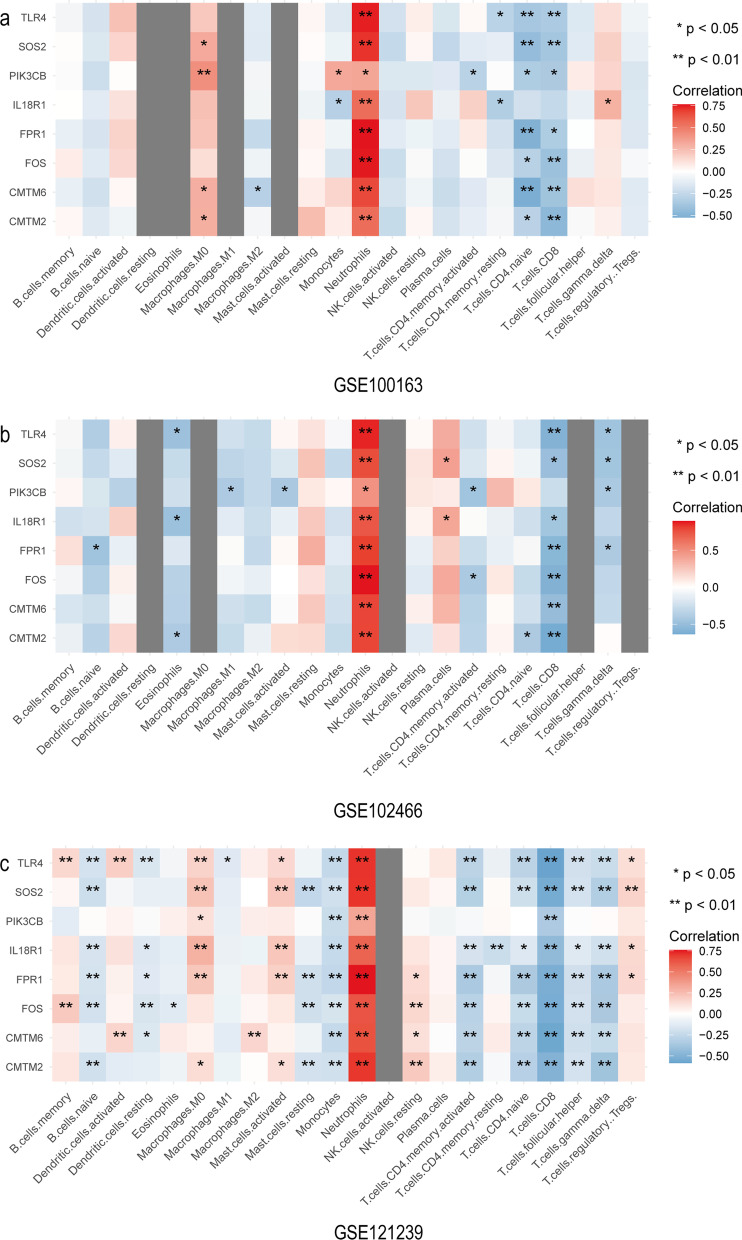
The correlation analysis results of eight biomarkers and 22 immune cells. The color shows the correlation coefficient; * marks those that are significant

The eight genes were associated with the abundance ratio of certain types of immune cells. As shown in Fig. 6, the colour shows the correlation coefficient, * marks those that are significant. The expression of the eight genes was significantly positively correlated with the neutrophil abundance ratio but inversely correlated with CD8 T cells and naive CD4 T cells (Fig. 6). These results suggest that the HUB genes may play a crucial role in immune cell function.

#### Eight biomarkers had a synergistic effect in the SLE-induced immune response

**Fig. 7:**
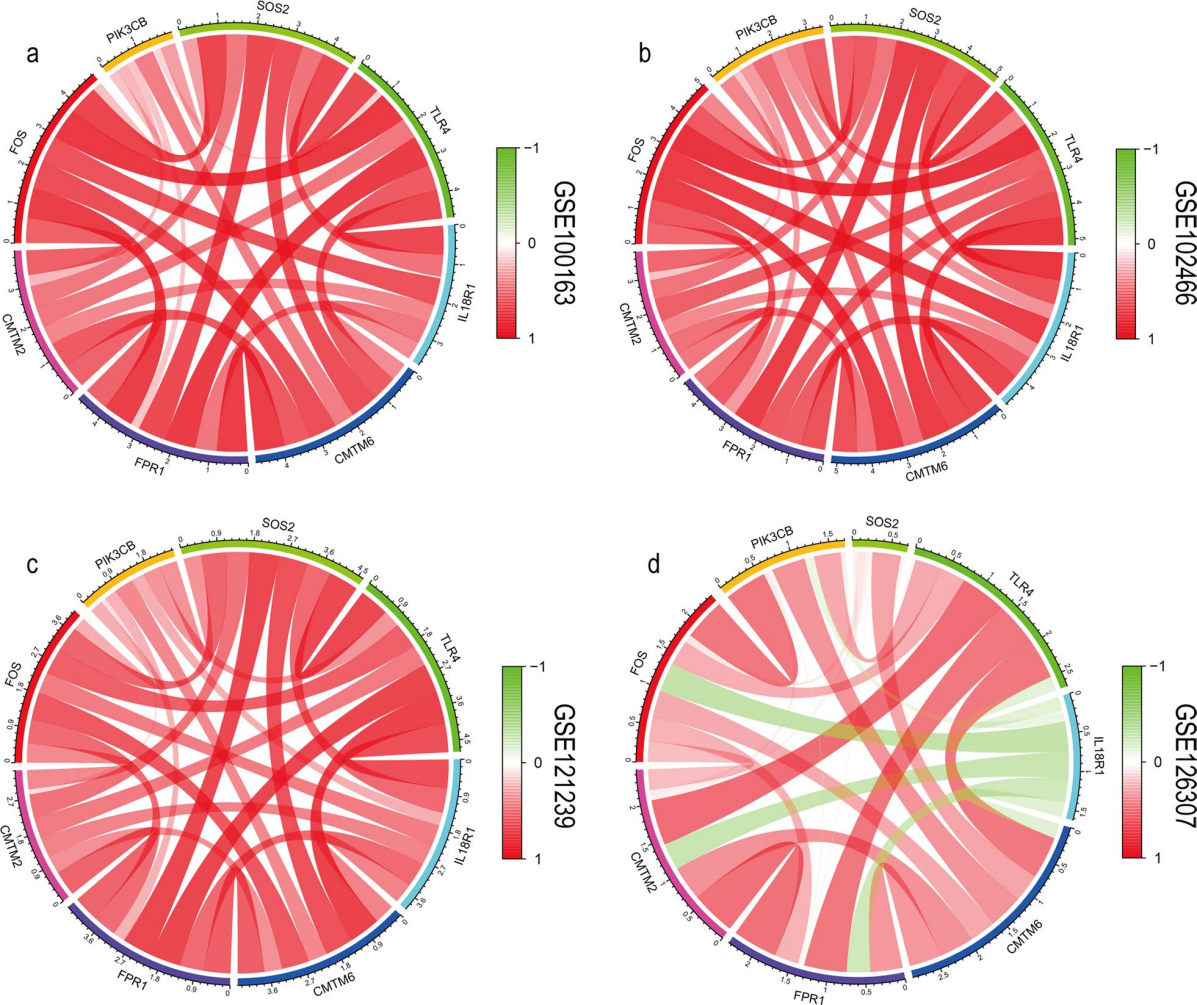
The relationship between each biomarker in GEO datasets. **a** GSE100163, **b** GSE102466, **c** GSE121239, **d** GSE126307

**Fig. 8:**
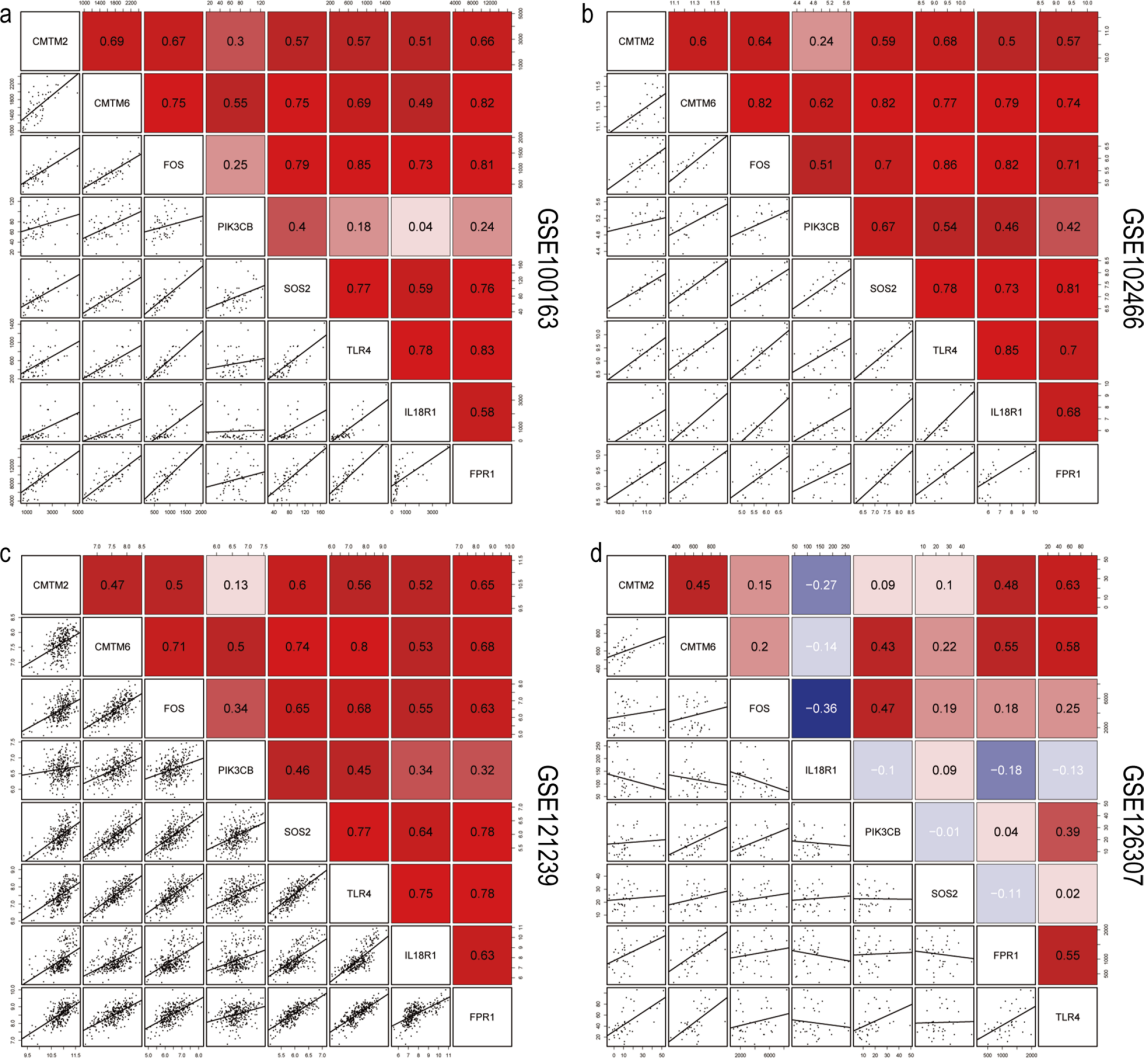
Scatter plot of gene correlation. Each dot represents a sample, and the black line represents the relationship between the expression level of genes. **a** GSE100163, **b** GSE102466, **c** GSE121239, **d** GSE126307

The correlation among the genes was evaluated to further explore the synergy of the eight genes in the immune response induced by SLE (Figs. 7, 8). In Fig. 7, red indicates a positive correlation and green represents negative correlation. Interestingly, *CMTM2*, *FOS*, *PIK3CB*, *SOS2*, *TLR4*, *IL18R1*, *CMTM6*, and *FPR1* were coregulated to regulate the SLE immune response, supporting the use of the biomarkers mentioned above in combination immunotherapy in future studies.

### Clinical factors and immune-related genes

**Fig. 9:**
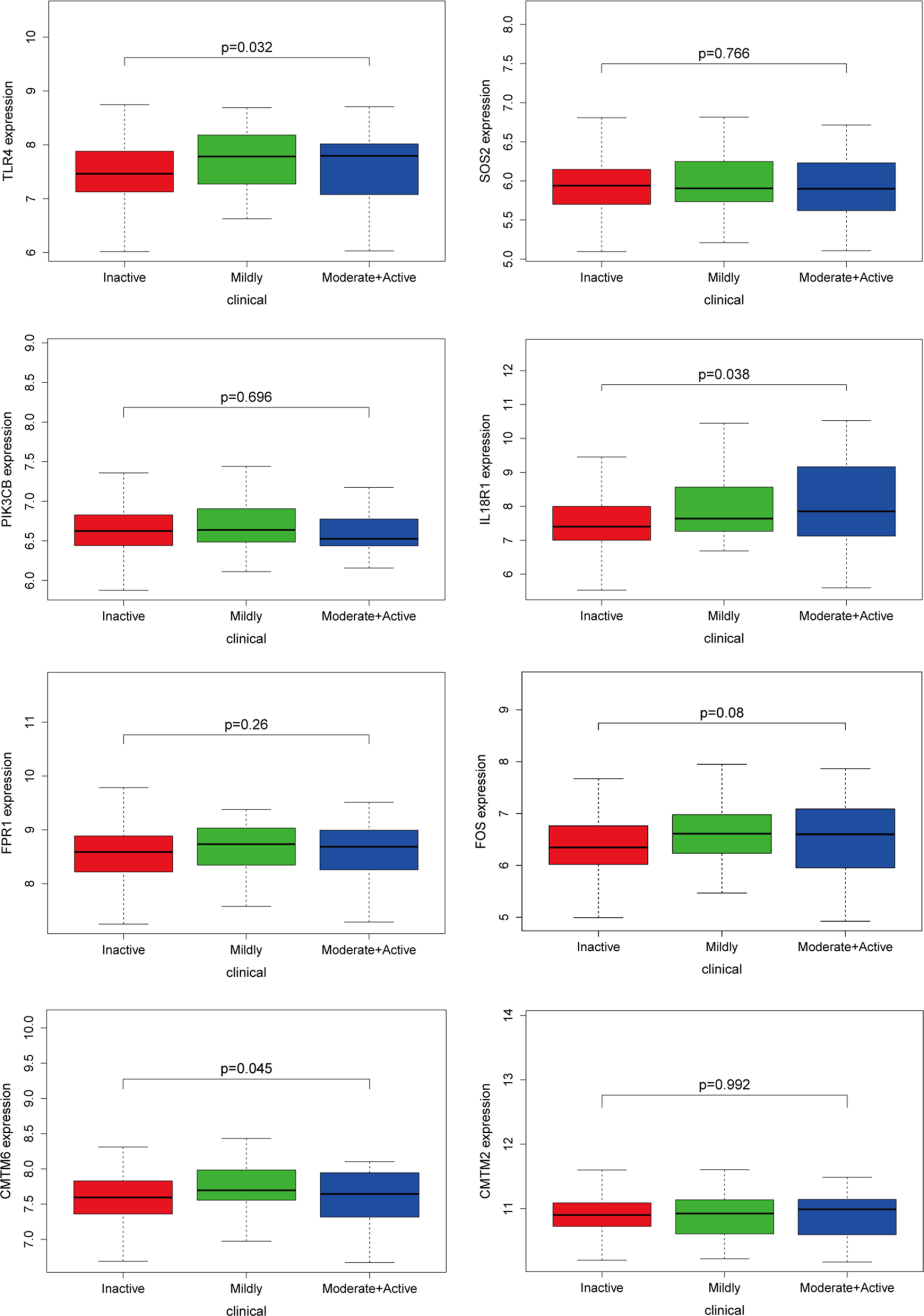
The correlationship between gene expression of the eight genes and SLE activity

Gene expression profiles and clinical information of 292 patients with SLE were downloaded from the GEO database. Clinical information included the patients’ systemic lupus erythematosus disease activity index (SLEDAI). The SLEDAI is a validated disease activity metric in which doctors assign scores to a list of 24 items, of which 16 are based on clinical symptoms and 8 are based on laboratory measurements of their presence or absence in the past 10 days [24]. More severe organ involvement, such as kidney involvement, was weighted with higher scores. These points were then added to possible scores ranging from 0 to 15, and the higher the score, the higher the disease activity. A score of 0–4 meant that the disease was basically inactive, a score of 5–9 meant that the disease was mildly active, a score of 10–14 meant that the disease was moderately active, and a score greater than or equal to 15 meant that the disease was severely active. Because the sample size of severe activity was small, moderate to severe activity was considered as a group, and the relationship between the expression of the eight genes and SLE activity was explored (Fig. 9). *TLR4*, *CMTM6*, *IL18R1*, and SLE activities were related (*P* < 0.05), and *IL18R1* expression increased with disease activity, indicating that *IL18R1* may be related to patients’ disease activity and/or unfavourable factors.

### GO and KEGG enrichment analyses

**Fig. 10:**
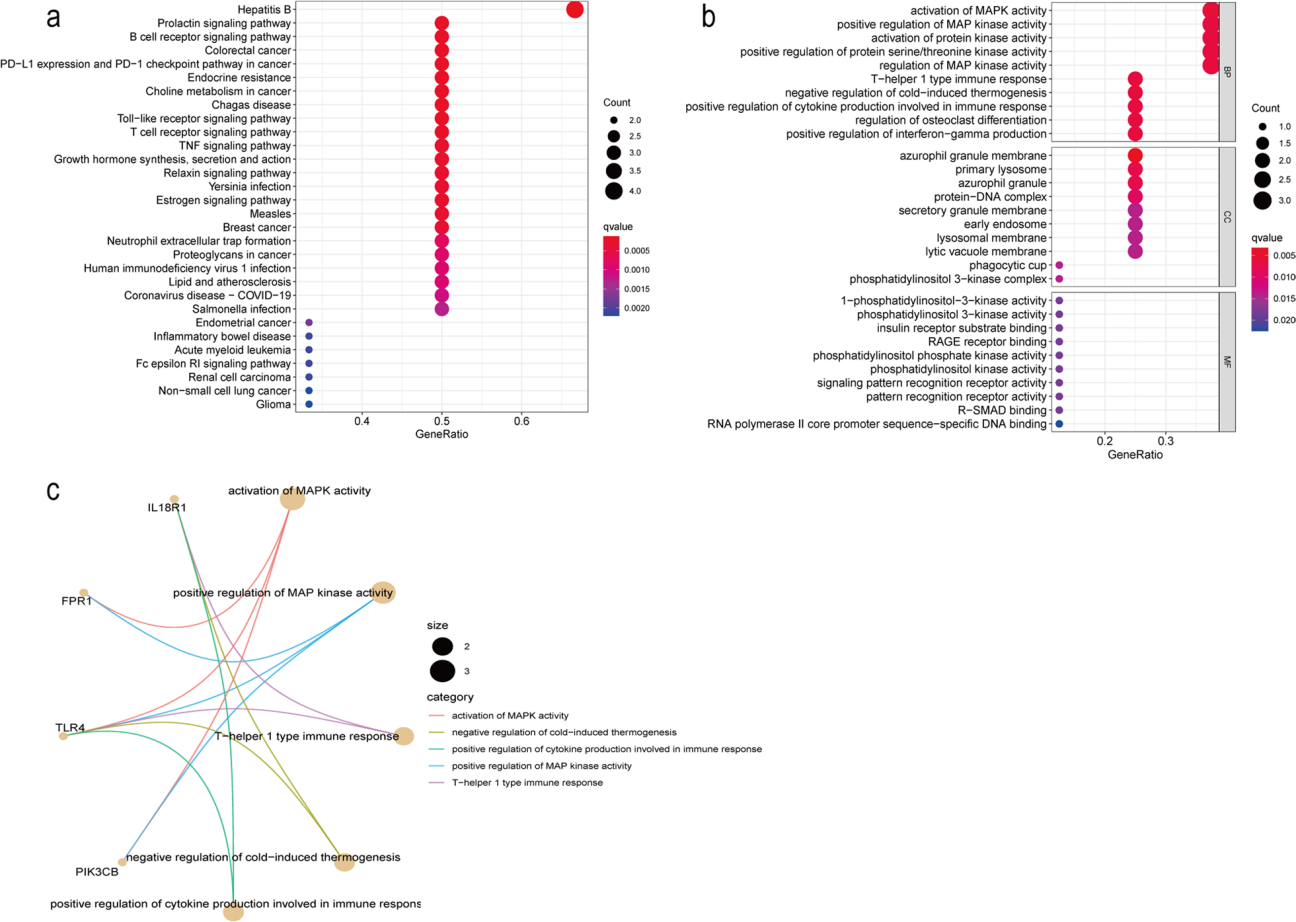
Analysis of the main mechanism of neutrophil-related biomarkers. **a** GO analysis of 8 kinds of neutrophil-related biomarkers. **b** KEGG analysis of neutrophil-related biomarkers. The significantly enriched pathways are shown. **c** Protein–protein interactions (PPIs) of hub genes

Eight neutrophil-specific hub genes were mainly enriched in BPs related to immune response and inflammatory response, including activation of mitogen-activated protein kinase (MAPK) activity, positive regulation of MAPK activity, positive regulation of immune response cytokine production, and positive regulation (Fig. 10). In the signalling pathway for interferon-gamma production, important CCs were azurophilic granules, primary lysosomes, and protein-DNA complexes. In addition, the hub genes participated in several signalling pathways related to the immune system, including B cell receptor signalling, Toll-like receptor (TLR) signalling, T cell receptor signalling, TNF signalling, and NET formation. These results are consistent with the available information of autoimmune diseases, including SLE, caused by abnormal neutrophils. In the PD-L1 signalling pathway, one of the novel enrichment pathways, D-1 (CD279) is an inhibitory receptor belonging to the CD28/cytotoxic T-lymphocyte-associated protein 4 families. Connections of PD-1 and its ligands PD-L1 (B7-H1; CD274) and PD-L2 (B7-DC; CD273) provide inhibitory signals leading to T-cell activation, tolerance, and immune-mediated tissue damage [25].

### GSEA based on SLE neutrophil subtype

**Fig. 11:**
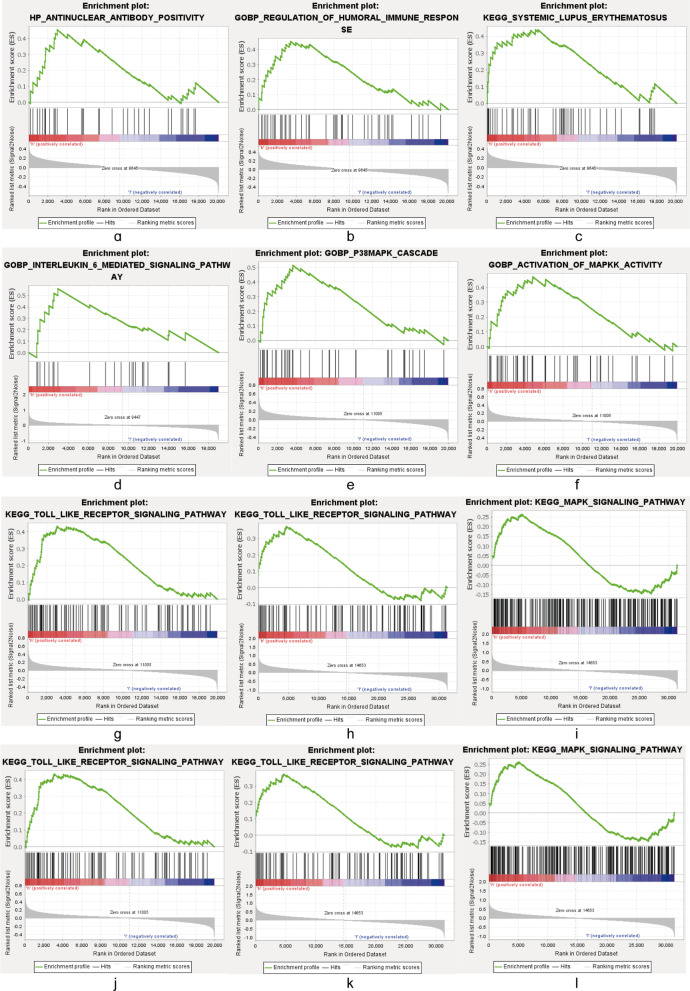
Gene set enrichment analysis (GSEA) was used to analyse the signal pathways enrichment in different subtypes. Several significant pathways were screened out

To explore the biological changes caused by differences in neutrophil enrichment, the GSEA results for each subtype were compared in pairs. As the enrichment of neutrophils increased, the production of antinuclear antibodies, interleukin (IL)-6-mediated signalling pathways, helper T cell differentiation, TLR binding pathways, and MAPK signalling pathways were upregulated. The results of this study suggest that the TLR-binding pathway and the MAPK signalling pathway were the two biological pathways most associated with neutrophil imbalance leading to SLE (Fig. 11).

### Drug prediction

**Fig. 12:**
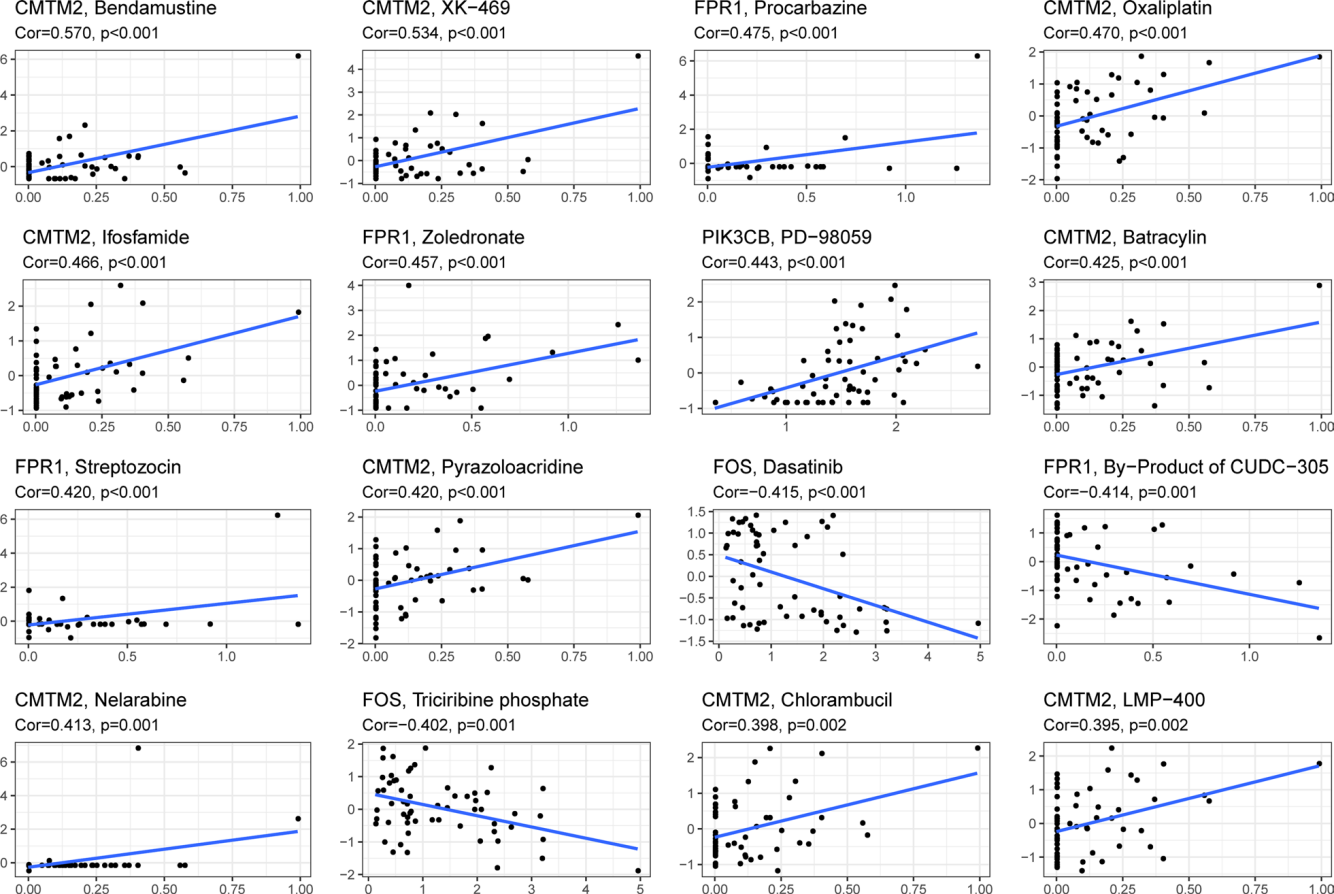
Correlation analysis between neutrophil-related biomarkers and drug sensitivity of drugs in CellMiner. The top 16 statistical significance correlation relationship between neutrophil-related biomarkers and drug sensitivity of drugs

*CMTM2* expression positively correlated with the sensitivity to chlorambucil, bendamustine, and ifosfamide (*P* < 0.001) (Fig. 12). The higher the *FOS* expression, the weaker the sensitivity to triciribine phosphate and dasatinib (*P* < 0.001). *PIK3CB* expression significantly positively correlated with the sensitivity of PD-98059 (*P* < 0.001).

## Discussion

SLE is a highly heterogeneous autoimmune disease, and patients with SLE are at a risk of progressive organ damage, reduced quality of life, and even death [26]. Current treatment goals for patients with SLE include long-term survival, prevention of sudden organ damage, and improvement of quality of life. For severe organ damage or life-threatening SLE, treatment usually consists of an initial stage of high-intensity immunosuppressive therapy to control disease activity, followed by longer-term and lower-intensity treatment to stabilise the condition and prevent recurrence [27].

With the advancement of genome-wide association studies and microarray technology, the understanding of gene expression has expanded, including gene expression related to SLE pathogenesis [28]. Nonetheless, the pathophysiological mechanism of SLE has not been thoroughly studied. A more detailed understanding of SLE pathogenesis promotes the progress of targeted therapies. Compared with that of tumours, the genotyping scheme for autoimmune system diseases is still in its infancy. To better diagnose and treat SLE, a unified, complete, and highly feasible molecular typing programme is needed. In addition, to date, no study has classified SLE based on the neutrophil genome [29]. To identify and classify reliable genes and pathways associated with SLE, a matrix was downloaded from the GEO database and analysed using comprehensive bioinformatic methods. Most previous health information analyses have focused on comparing patients with SLE with healthy people. This study only involved patients with SLE, mainly based on the GEO, immunological (ImmPort), SSGSEA, WGCNA, consensus clustering, and CELLMINER databases. First, neutrophil-related biomarkers were identified using the WGCNA. The eight genes (*CMTM2*, *FOS*, *PIK3CB*, *SOS2*, *TLR4*, *IL18R1*, *CMTM6*, and *FPR1*) that were highly related to neutrophils may mediate SLE development, and they are potential targets and biomarkers for disease treatment and SLE activities.

Subsequently, a specific neutrophil genome was used to identify and verify this new SLE classification. SLE was divided into two stable subtypes, namely, NEUT_H and NEUT_L. CIBERSORT was used to analyse the immune cells of neutrophil subtypes; there was less CD8 T cell infiltration in the NEUT_H group. The reduction of CD8 T cells may impair T cell ability to cope with infection in patients with SLE, further aggravating SLE [30]. Next, the biological changes caused by the enrichment of neutrophils at different levels of SLE were explored; the results indicated that NEUT_H and antinuclear antibody formation, IL-6-mediated signalling pathways, helper T cell differentiation, TLR body binding pathway, and the MAPK signalling pathway positively correlated. NETs play a key role in SLE pathogenesis [31]. Activation of the MAPK signalling pathway and IL-6-mediated signalling pathway, and excessive proinflammatory cytokines also lead to increased NET formation, promoting tissue damage and inflammation [32]. Inhibiting NET formation may be a new target for SLE treatment [33]. In this study, we applied bioinformatic methods to identify genes related to the formation of NETs from the genetic perspective and to provide new treatment ideas.

As an important receptor for neutrophils to recognise pathogens, TLRs affect phagocytosis and neutrophil sterilisation, as well as other related immune functions [34]. Over-activated TLRs may induce an excessive immune response, adversely affecting SLE occurrence. TLR4 is a key component of innate and adaptive immune responses during infection and inflammation [35]. The pathogenic role of TLR4^+^ plasma cells in lupus nephritis development is well known. In addition, in vivo treatment with TLR4 inhibitors significantly reduces the production of autoantibodies and kidney damage in mice with SLE [36]. It has also been observed that TLR4 expression in neuropsychiatric lupus increases [37]. The present study demonstrated that the role of TLR4 in the pathogenesis of SLE disease might be through the suppression of regulatory T cells and the effect of proinflammatory cytokines (such as IL-6 and TNF-α) on SLE pathogenesis, inducing TLR4-mediated neutrophil activation. This study also provided more evidence for the advancement of TLR4 inhibitors in SLE treatment.

The CMTM family is widely expressed in the immune system [38]. Abnormal expression of CMTM is related to the occurrence and development of various diseases; both CMTM4 and CMTM5 are involved in SLE pathogenesis [22]. Existing studies have shown that the CMTM family has considerable clinical value in understanding the pathological stages of breast cancer, non-small cell lung cancer, and gastric cancer, determining treatment strategies, and predicting prognosis. *CMTM6* may act as an oncogene in various solid tumours [39]. Studies have shown that neutrophils are involved in antiphospholipid syndrome (APS) pathogenesis. NETs play an important role in APS because neutrophils in patients with APS are prone to spontaneously release NETs. Knight et al. found that CMTM6 is upregulated in the neutrophils of patients with APS [40, 41]. The current results indicated that *CMTM2* and *CMTM6* might be related to NET formation through their upregulation in patients with SLE, leading to disease occurrence, and they might be used as new therapeutic targets.

IL18R1 encodes the α subunit of the IL-18 receptor and is responsible for the binding of IL-18; the activated receptor then initiates the same signalling pathway as IL-1 to activate nuclear factor (NF)-κB [42]. The NF-κB signalling pathway includes the classic NF-κB activation pathway and the alternative pathway, and these two pathways play an important role in regulating immune and inflammatory responses. In human and mouse experiments, high plasma levels of B cell-activating factor may trigger the non-classical NF-κB signalling pathway [43]. The extracts of *Tripterygium wilfordii* Hook F, demethylzeylasteral (T-96), and methylprednisolone (MP) have been shown to improve MRL/lpr susceptibility to SLE by inhibiting the NF-κB signalling pathway in rats with lupus nephritis [44, 45]. The present study confirmed that IL18R1 overexpression leads to the NF-κB signalling pathway activation, driving disease activity. *IL18R1* expression increased with disease activity, indicating that IL18R1 might be an unfavourable factor for disease activity in patients with SLE. Thus, monitoring *IL18R1* expression may help to better assess SLE activity.

A recent study showed that the formyl peptide receptor FPR2 is abundantly expressed by neutrophils, where it regulates the recruitment of proinflammatory tissues of inflammatory cells, production of ROS, and regression of inflammatory responses [46]. FPR treatment is used as a new type of anti-inflammatory therapy [46].

At present, the standard SLE treatment includes non-steroidal anti-inflammatory drugs, glucocorticoids, hydroxychloroquine, and non-specific immunosuppressive agents [47]. The CellMiner database contains genome and pharmacological tools to identify drug patterns and transcripts in the NCI-60 cell line [48]. The CellMiner database compares the expression levels of 360 microRNAs, 22,379 genes, and 20,503 compounds, including 102 FDA-approved drugs [1]. In this study, we first analysed the relationship between the hub genes and drug sensitivity and provided new insights to explore the choice of SLE immunosuppressants and avoid drug resistance. Chlorambucil, an immunosuppressant with a suppressive effect on human bone marrow and usually combined with hormonal drugs to effectively alleviate nephritis symptoms, may selectively be used for SLE treatment [49]. PD 98059 prevents the MAPK cascade (MKK1) by blocking the MAPK cascade reaction, as determined using MAPK-based kinase assays, instead of directly inhibiting MKK1 activity [50], further predicting relevant drugs for SLE treatment.

In summary, pivotal genes associated with neutrophils in patients with SLE were identified, and patients were classified into two different subtype groups. Biomarkers that may be related to SLE activity were explored. Using internal verification, the versatility of these biomarkers was demonstrated. The functional analysis emphasised the importance of the obtained biomarkers because they participate in several important pathways. However, this study had some limitations. First, a multi-centre prospective study is required to evaluate the utility of neutrophil typing in patients with SLE. Second, in vivo and in vitro experimental verification is warranted to clarify the underlying molecular mechanism. Finally, analysis of the survival rate of patients with SLE is lacking.

## Conclusions

Based on multiple dataset analyses, a molecular classification of SLE was established based on neutrophil-related subtypes, more comprehensively demonstrating the clinical characteristics of subtypes, immune infiltration, the relationship between the corresponding pathways and key genes, and the effect of immunotherapy. In view of the limited samples for SLE classification based on neutrophil characteristics, a larger sample size analysis and further basic experiments are needed to support this pioneering classification. Nevertheless, the current in-depth analysis of neutrophil-related biomarkers yields valuable references and insights and provides evidence to develop new strategies for the precise treatment of SLE.

## Supplementary Information

**Additional file 1**. 1671 immune genes which were downloaded from the IMMPORT database.

**Additional file 2**. Co-expression modules results of datasets.

**Additional file 3**. GO and KEGG gene sets used in the GSEA.

**Additional file 4**. Grouping of GSEA.

**Additional file 5**. Input data sets of immune cell infiltration.

**Additional file 6**. Pearson value between netrophil-related genes.

**Additional file 7**. Results of inmmune cell infiltration.

**Additional file 8**. Scripts used for immune cell infiltration.R.

**Additional file 9**. Scripts used for immune cell infiltration.R

**Additional file 10**. Module–trait relationships in SLE of WGCNA.

**Additional file 11**. The relative immune cell abundance of single-sample gene set enrichment analysis.

## Data Availability

All data generated or analysed during this study are included in this published article and its supplementary information files. The datasets analysed during the current study are available in the Gene Expression Omnibus (GEO) repository, https://www.ncbi.nlm.nih.gov/geo/.

## Abbreviations

APS: Antiphospholipid syndrome
AUC: Area under the curve
BP: Biological process
CC: Cell component
CDF: Cumulative distribution function
FDA: Food and Drug Administration
GEO: Gene Expression Omnibus
GO: Gene Ontology
GS: Gene significance
GSEA: Gene set enrichment analysis
KEGG: Kyoto Encyclopedia of Genes and Genomes
LDG: Low-density granulocyte
MAPK: Mitogen-activated protein kinase
ME: Module eigengene
MF: Molecular function
MM: MODULE membership
NET: Neutrophil extracellular traps
PPI: Protein interaction
ROS: Reactive oxygen species
SLE: Systemic lupus erythematosus
SLEDAI: Systemic lupus erythematosus disease activity index
TLR: Toll-like receptor
