# Supplementary material for: Identification of biomarkers related to neutrophils and two molecular subtypes of systemic lupus erythematosus
Source: BMC Med Genomics. 2022 Jul 20;15:162. doi: 10.1186/s12920-022-01306-9 (PMC9297641; doi:10.1186/s12920-022-01306-9)
Supplement: Supplementary file 10 — Additional file 10. Module–trait relationships in SLE of WGCNA. [file 12920_2022_1306_MOESM10_ESM.docx]

Supplementary material 1: Module–trait relationships in SLE. All cell types were based on the identification of key genes in the SLE neutrophil subtype of WGCNA.
